# Exploring the temporal structure of heterochronous sequences using TempEst (formerly Path-O-Gen)

**DOI:** 10.1093/ve/vew007

**Published:** 2016-04-09

**Authors:** Andrew Rambaut, Tommy T. Lam, Luiz Max Carvalho, Oliver G. Pybus

**Affiliations:** ^1^Institute of Evolutionary Biology,; ^2^Centre for Immunity, Infection and Evolution, University of Edinburgh, Ashworth Laboratories, King’s Buildings, Edinburgh EH9 3JT, UK,; ^3^School of Public Health, University of Hong Kong, Hong Kong SAR, China and; ^4^Department of Zoology, University of Oxford, South Parks Road, Oxford OX1 3PS, UK

**Keywords:** phylogeny, molecular clock, evolutionary rate, regression, model selection

## Abstract

Gene sequences sampled at different points in time can be used to infer molecular phylogenies on a natural timescale of months or years, provided that the sequences in question undergo measurable amounts of evolutionary change between sampling times. Data sets with this property are termed heterochronous and have become increasingly common in several fields of biology, most notably the molecular epidemiology of rapidly evolving viruses. Here we introduce the cross-platform software tool, TempEst (formerly known as Path-O-Gen), for the visualization and analysis of temporally sampled sequence data. Given a molecular phylogeny and the dates of sampling for each sequence, TempEst uses an interactive regression approach to explore the association between genetic divergence through time and sampling dates. TempEst can be used to (1) assess whether there is sufficient temporal signal in the data to proceed with phylogenetic molecular clock analysis, and (2) identify sequences whose genetic divergence and sampling date are incongruent. Examination of the latter can help identify data quality problems, including errors in data annotation, sample contamination, sequence recombination, or alignment error. We recommend that all users of the molecular clock models implemented in BEAST first check their data using TempEst prior to analysis.

## 1. Introduction

Gene sequences are denoted ‘heterochronous’ (or measurably evolving) if they have been obtained from natural populations at evolutionarily distinct points in time. In this context, two sampling times are ‘evolutionarily distinct’ if genetic sequences sampled at those times differ by a measurable amount of nucleotide or amino acid substitution within the sampled population (Drummond et al. 2003a). Such data sets have become increasingly common in a range of biological disciplines, including infectious disease epidemiology, molecular ecology, molecular taxonomy, archaeology, and anthropology (e.g., Willerslev and Cooper 2005; Pybus and Rambaut 2009; Biek et al. 2015). In the past, most heterochronous data sets comprised gene sequences from either RNA viruses or ancient DNA studies of animal populations. Many RNA viruses evolve so rapidly that sequences sampled only weeks or months apart may be evolutionarily distinct, whereas ancient DNA sequences can be recovered from preserved biological material many thousands of years old, such that they are genetically different from those obtained from contemporary animal populations. More recently the concept of heterochronous data has been extended to slower-evolving micro-organisms, including DNA viruses (e.g., Firth et al. 2010) and bacteria (e.g., Lowder et al. 2009), in part as a result of the increasing availability of whole-genome sequences for these species (Biek et al. 2015). It seems very likely that heterochronous data sets will continue to grow in popularity as sequencing technologies increase in power and decline in cost.

The evolutionary and phylogenetic analysis of gene or genome sequences from different points in time necessitates the use of methods distinct from those typically applied to ‘isochronous’ data sets (i.e., alignments that contain sequences sampled simultaneously, or over a time range whose duration is trivial compared to the evolutionary timescale of the species under investigation). Most importantly, the sampling dates of heterochronous sequences contain information about the rate of sequence evolution and consequently such data sets can be used to directly infer molecular phylogenies on a natural timescale of months, years, or millennia. By contrast, the branches of phylogenies estimated from isochronous data sets represent genetic distance only, and the independent effects of evolutionary rate and divergence time on genetic distances cannot be separated without external information about one or the other. Phylogenies whose branch lengths represent time (‘time trees’ or ‘clock trees’) have advantages over those measured as genetic distance, as the timescale provides a common frame of reference that enables evolutionary change to be directly compared with known historical events. For example, Shapiro et al. (2004) used heterochronous ancient mtDNA sequences sampled across a period of 60,000 years to suggest that the rapid decline in the genetic diversity of North American bison began before, not after, the first evidence of human hunters in the region.

In order to estimate phylogenies (and other evolutionary parameters, such as effective population sizes or speciation rates) on a natural timescale of years requires a ‘molecular clock’ model, which, in essence, is a statistical description of the relationship between observed genetic distances and time. The early development of the ‘molecular clock’ concept is intertwined with historical debates over the applicability of Kimura’s Neutral Theory of Evolution to empirical data (e.g., Gojobori, Moriyama, and Kimura 1990). However, it is important to note that it is not necessary to assume the absence of natural selection in order to infer phylogenies on a natural timescale. A suite of models, generally referred to as *relaxed* or *local* molecular clocks, have been developed that allow the rate of evolution to vary (for whatever reason) among the branches of a phylogenetic tree (e.g., Huelsenbeck, Larget, and Swofford 2000; Kishino, Thorne, and Bruno 2001; Drummond et al. 2006; Drummond and Suchard 2010). Time-scaled trees can be estimated using many statistical approaches, including Bayesian inference (e.g., Drummond et al. 2012), maximum likelihood (e.g., Rambaut 2000; Sanderson 2003; Yang 2007), or heuristic methods (e.g., Drummond and Rodrigo 2000).

Before using a molecular clock model to infer a time-scaled tree from heterochronous sequences, it is advisable to confirm that the sequences under investigation contain sufficient ‘temporal signal’ for reliable estimation. In other words, there must be sufficient genetic change between sampling times to reconstruct a statistical relationship between genetic divergence and time. This is particularly important for Bayesian inference approaches such as those implemented in BEAST, because the molecular clock models employed are statistically conditioned on having an evolutionary rate greater than zero, and will usually allow inference to proceed even when the alignments being analysed contain little or no temporal information. In such cases, the software may give the appearance of a statistically well-supported timescale even when none exists. Ideally, in such circumstances, the posterior estimates of the rate of evolution should reflect the prior distribution, but in reality random error and model misspecification may result in misleading conclusions being drawn.

It is therefore important for researchers to explore the degree of temporal signal in heterochronous sequences *before* proceeding to inference using formal molecular clock models. This can be achieved using a simple regression-based approach. Suppose we have a rooted molecular phylogeny (whose branch lengths represent genetic distance) estimated from heterochronous sequences. For the moment we will assume the tree is rooted correctly. For each sequence *i* let *t_i_* be the sampling time of that sequence, and let *d_r,i_* be the genetic distance between that tree tip and the tree root (the so-called ‘root-to-tip’ distance). If all branches evolve at the same rate (i.e., according to a strict clock), then the phylogenetic timescale can be estimated using the following linear regression model

(1) $$\text{E}[d_{r,i}]=u\left(t_{i}–t_{r}\right),$$

where *u* is the rate of sequence evolution and *t_r_* is the time of the tree root. If *d_r,i_* is plotted against *t_i_*, then the gradient of the regression line represents *u* and the *x*-intercept represents *t_r_* (Buonagurio et al. 1986; Gojobori, Moriyama, and Kimura 1990; Drummond, Pybus, and Rambaut 2003b). A heterochronous alignment with strong temporal information will generate a notable correlation between *d_r,i_* and *t_i_* (interpretation of the statistical significance of this regression is not straightforward; see below).

Regression of root-to-tip genetic distance against sampling time can be used as a simple diagnostic tool for molecular clock models. Specifically, a linear trend with small residuals indicates that evolution will be adequately represented by a strict molecular clock. The same trend with greater scatter from the regression line suggests a relaxed molecular clock model may be most appropriate. A strong non-linear trend suggests that evolutionary rate has systematically changed through time, whereas no trend at all indicates that the data contain little temporal signal and is unsuitable for inference using phylogenetic molecular clock models. Evidence for local clock models may be found by selecting tree tips that correspond belong to a specific phylogenetic group or lineage, then visualizing the position of the regression points that correspond to the selected taxa.

In addition to the problem of poor temporal signal, molecular clock analyses can be hampered by data quality issues. Annotation errors can be introduced into large sequence alignments and sequences obtained from GenBank or other public databases may be labelled with incorrect sampling dates. A further problem present in some viral or bacterial sequences alignments is the possible inclusion of laboratory or vaccine reference strains that have spent many years in frozen storage. From a molecular clock perspective, the ‘correct’ sampling date of these is the date they were placed in storage, not the date they were ultimately sequenced. Inference methods such as those implemented in BEAST are dependent upon the sampling dates provided by the user and thus results will be affected by data quality issues without giving any indication that they might be present (e.g., Graur and Pupko 2001; Worobey 2008). Fortunately, the regression approach outlined above can be also used as a quality control step before undertaking computationally intensive phylogenetic analyses.

Although regressions of sampling time versus root-to-tip genetic distance can be used to investigate temporal signal and data quality in heterochronous alignments, they are not suitable for statistical hypothesis testing because the individual data points are not independently distributed, and are instead partially correlated due to their phylogenetic shared ancestry. This effect is particularly strong if sequences from different sampling times are genetically distinct from one another (Murray et al. 2016). Therefore the *r*^2^ value, P-value, and parameter confidence limits of the regression model are not valid statistical estimates (see Drummond et al. 2003a for further discussion). The regression should therefore be used as a data exploration tool rather than for formal hypothesis testing.

## 2. TempEst

The program TempEst (TEMPoral Exploration of Sequences and Trees) is a cross-platform, open source, graphical program for exploring heterochronous data, freely available from http://tree.bio.ed.ac.uk/. During its development TempEst was formerly known as Path-O-Gen. Its name has been changed to reflect the fact that the software can be applied to all heterochronous alignments, not just those from pathogenic micro-organisms.

**Figure 1.**
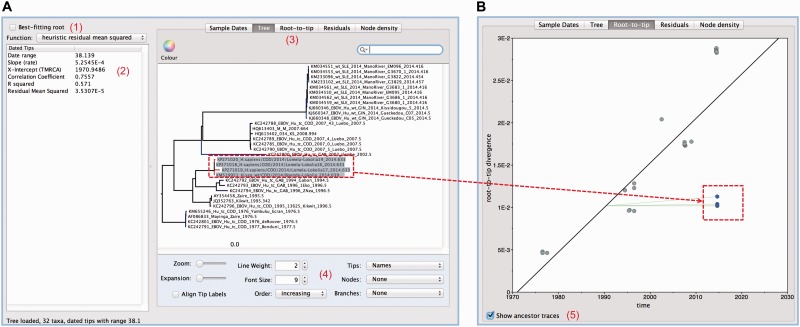
User interface of TempEst. (A) The ‘tree’ panel and (B) the ‘root-to-tip’ regression panel. If a user selects a taxon or group of taxa in one panel, then the corresponding sequences or points will be highlighted in other panels (e.g., the four taxa highlighted in the tree panel are shown in blue in the root-to-tip panel). Components of the user interface discussed in the text are highlighted. (1) Button that initiates estimation of the best-fitting root location. (2) Regression analysis parameter estimates. (3) Tabs to switch between different data visualization panels. (4) Options to adjust how the tree is displayed. (5) Option to show ancestor traces (thin green lines). Ancestor traces for a subset of taxa are also shown if some taxa are highlighted.

As input, TempEst takes a ‘non-clock’ phylogenetic tree (i.e., one whose branch lengths are scaled as genetic distances), which can be estimated using standard neighbour-joining, maximum likelihood, or Bayesian approaches (Felsenstein 2003). Once loaded, the user provides sampling dates or ages for each sequence. The dates can be manually entered, extracted from fields embedded in the sequence labels, or loaded from a tab-delimited table. The ‘Root-To-Tip’ analysis panel (Fig. 1) is then displayed, showing a linear regression of root-to-tip genetic distance against sampling time for each tip (see Equation 1). Various parameters are presented in a table to the left of the plot, including an estimate of the rate of evolution (i.e., the gradient of the regression), and the intercept with the time-axis, which is a valid point estimate of the age of the phylogeny root (Fig. 1).

When a phylogeny is initially loaded, TempEst assumes that the tree is rooted at a position chosen by the user. However, most methods for reconstructing ‘non-clock’ phylogenies deliver a tree that is arbitrarily rooted, so the user should actively root the tree before loading it into TempEst. Alternatively, TempEst provides an option to locate a ‘best-fit’ root position. If this option is selected, then TempEst finds the root location that minimizes the sum of the squared residuals from the regression line. Although other criteria have been employed to find a best-fit root (Drummond et al. 2003a; Maljkovic Berry et al. 2009) we have found, anecdotally, that minimizing the residuals is a robust criterion under moderate levels of among-branch rate heterogeneity. Although this procedure finds the root for which the data appear most ‘clock-like’, it does allow the possibility of a negative slope being offered. This is not a problem, because the estimation of a negative evolutionary rate indicates that the data set contains little or no temporal signal.

TempEst provides two further data views. The ‘Tree’ analysis panel shows the phylogeny itself. External phylogeny branches are coloured according the position of their respective tips in the corresponding regression plot. By default, blue branches represent points below the regression line, indicating sequences that are less divergent (for their sampling date) than average. Red branches represent the opposite situation. The ‘Residuals’ analysis panel contains a histogram and scatterplot of the residuals of the linear regression. Importantly, all four analysis panels are linked, so that data points (tree tips) selected in one panel are automatically highlighted in the other two (see Fig. 1). This enables easy investigation of outliers and sequences or clades of interest.

TempEst can also be used to explore isochronous phylogenies, that is, those whose tips are sampled at the same time. In such cases, the sampling date input step is omitted and the ‘Root-to-tip’ panel presents a histogram of the genetic distance of each tip from the tree root. As the amount of time between the root and each tip in an isochronous tree is identical, this plot gives a measure of the variation in evolutionary rate across the tree (although, as explained above, this measure is not suitable for hypothesis testing because the regression points are not statistically independent). If the ‘best-fitting root’ option is selected when an isochronous tree is loaded, then TempEst will attempt to find the root that minimizes the variance of root-to-tip distances.

### 2.1 Example 1: exploring temporal signal

**Figure 2.**
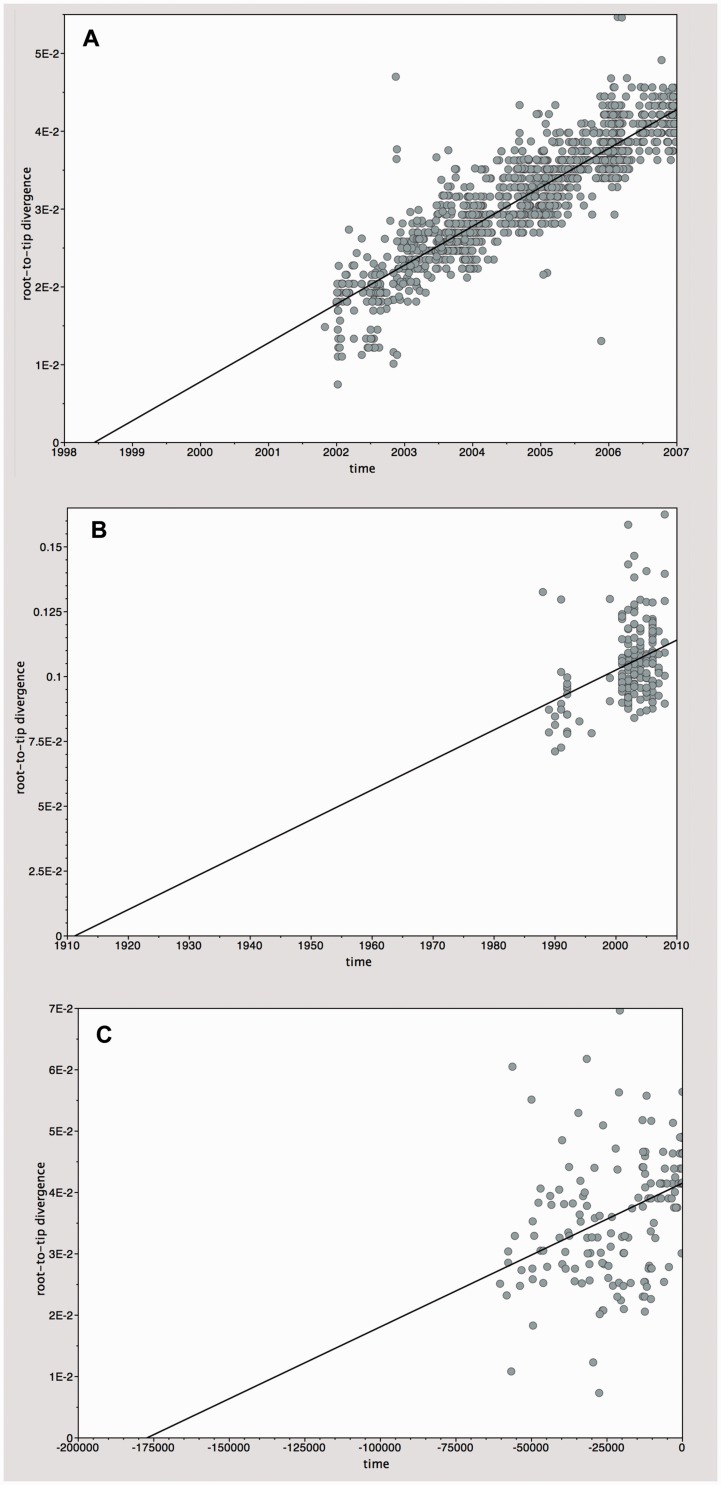
Root-to-tip regression analyses. Plots of the root-to-tip genetic distance against sampling time are shown for phylogenies estimated from three alignments: (A) 1,441 HA gene sequences belonging to seasonal human influenza A/H3N2 virus, sampled between 2001 and 2006. (B) Whole-genome sequences of 167 HCV subtype1b strains, sampled between 1988 and 2008. (C) A mtDNA control region fragment from 182 bison samples, sampled from >60,000 years before the present to the present day (time = 0). Sampling dates are given as years before the present.

Here we use three example data sets to illustrate how TempEst allows users to explore temporal signal in sequence alignments. The three data sets comprise: (1) haemagglutinin gene sequences of human influenza A/H3N2 viruses (Russell et al. 2008), (2) whole-genome sequences of the hepatitis C virus, and (3) modern and ancient mtDNA sequences obtained from bison, recovered from preserved biological samples of varying ages, the oldest being >60,000 years before present (Shapiro et al. 2004). Figure 2 shows the regressions of root-to-tip genetic distance against sampling time for each of these four data sets. This is what users see in the ‘Root-to-tip’ panel of TempEst (Fig. 1).

The three data sets demonstrate different levels of temporal signal, as assessed by visual inspection or by the correlation coefficient, *R*^2^. The *R*^2^ value can be used as an informal measure of dispersion around the best-fit line but should not be used to test the statistical significance of the regression (Drummond, Pybus, and Rambaut 2003b; see above). The human influenza virus phylogeny (Fig. 2A) exhibits the strongest association between genetic distances and sampling dates (*R*^2 ^= 0.80). The bison mtDNA (Fig. 2C) and hepatitis C virus (Fig. 2B) trees have more diffuse regression plots (*R*^2 ^= 0.21 and *R*^2 ^= 0.13, respectively). All data sets exhibit a positive correlation between genetic divergence and sampling time and appear to be suitable for phylogenetic molecular clock analysis in BEAST or other programs. Formal model selection (undertaken using likelihood ratios or Bayes Factors, e.g.) will be necessary to determine, for each data set, whether a strict, relaxed or local clock model is the most appropriate model for analysis.

### 2.2 Example 2: heterochronous data quality control

**Figure 3.**
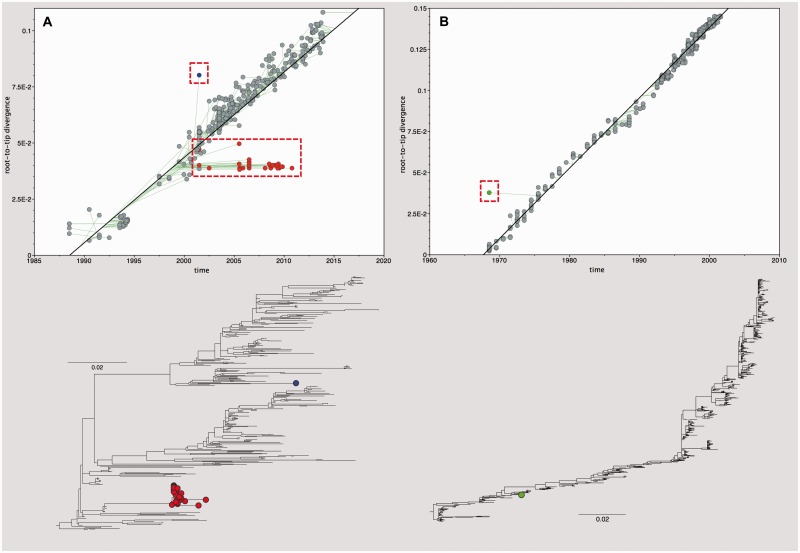
Heterochronous data quality control. (A) Root-to-tip regression plot, with ancestor traces shown, of 355 HA gene sequences belonging to the Classical swine lineage of influenza A/H1 virus. The outlier (blue circle) is strain A/Swine/North Carolina/98225/01(AF455676) which is thought to be a recombinant sequence (Lam et al. 2013). The group of outliers below the regression line (red circles) represents a vaccine lineage that has spent time in laboratory storage before resuming onward transmission (e.g., EU502884, DQ058215, HQ541680). Hence, this lineage has undergone less divergence from the tree root than expected. (B) Root-to-tip regression plot, with ancestor traces shown, of 614 HA gene sequences of human influenza A/H3N2 virus. The outlier (green circle) is strain A/Victoria/1968 (CY015508). This sequence has been retracted from GenBank, possibly the strain information does not match the sequence given, which appears to be of more recent provenance. Phylogenies for each regression plot are shown below, with outliers highlighted (scale bar represents substitutions per site).

TempEst can help to identify sequences whose sampling date is incongruent with their genetic divergence and phylogenetic position, and can provide clues as to the potential cause of such anomalies. Investigation begins by selecting the ‘Show ancestor traces’ button in the ‘Root-to-tip’ panel. Once activated, this tool draws a line from each data point on the regression plot (i.e., each tip) to a position on the regression line; the latter is determined by the root-to-tip genetic distance of the phylogenetic node that is immediately ancestral to the selected tip (Fig. 3). This allows the user to distinguish between two situations:

- A sequence/tip lies substantially above (or below) regression line (large *y*-axis residual). This indicates that the tip has considerably more (or less) genetic divergence from the root than one would expect given its date of sampling. This scenario is illustrated in Fig. 3A by the tip highlighted in blue. This situation may indicate a problem with the sequence itself, such as (1) low sequencing quality, (2) errors in sequence assembly, (3) an alignment error in part of the sequence, (4) an error in phylogenetic inference, (5) excessive passaging of a micro-organism leading to the accumulation of cell-line adaptations, or (6) a biological process such as recombination or hypermutation.
- When a sequence/tip lies substantially to the left or right of the regression line (large *x*-axis residual). This indicates that the specified date of sampling does not match the observed genetic divergence and is illustrated in Fig. 3B by the tip highlighted in green. Possible causes of this situation include (1) sequences that are mislabelled and have been ascribed an incorrect date of sampling during data collation and processing, (2) biological contamination by a sample from a different time, (3) an error in phylogenetic inference, and (4) the use of incorrect sampling dates for archived, reference or vaccine virus strains. An example of the latter situation is given in Fig. 3A (red points); vaccine or laboratory reference strains undergo little to no evolution while in storage and therefore their root-to-tip divergence is lower than their sampling date would suggest.

## 3. Discussion

The phylogenetic molecular clock methods implemented in BEAST and other software packages are powerful and often easy to use, but they make significant biological assumptions and are dependent on the quality of the sequence data used. We recommend that TempEst is always used to investigate heterochronous sequence alignments *before* they are subjected to model-based inference in BEAST. We hope that by using the tools implemented in TempEst researchers will avoid reporting evolutionary rate estimates that are either invalid (due to an absence of temporal signal) or biased (due to the inclusion of sequences with wrong sampling dates, alignment errors, etc.). As with all phylogenetic methods, TempEst assumes that recombination is rare or absent within the sample under investigation, although, as noted above, it may sometimes detect isolated recombinant sequences within an alignment of non-recombinant sequences.

TempEst is a tool for qualitative data exploration and should not be used to test hypotheses or undertake statistical model selection. Because sequences are statistically non-independent, these tasks should be performed in a phylogenetic framework that explicitly incorporates shared ancestry. Research is ongoing into the development of statistical methods that can formally test for the presence of temporal signal in heterochronous alignments. Drummond et al. (2003a) sketched, in the context of two sequences, a simple likelihood ratio test of the null hypothesis that there is zero measurable molecular evolution between sampling times. Firth et al. (2010) introduced an alternative randomization approach. Specifically, sampling dates are randomized on tree tips multiple times, then each randomization replicate is subjected to phylogenetic molecular clock inference using BEAST. The distribution of rates estimated from the randomization replicates is then compared to the estimate obtained from the empirical data (Firth et al. 2010). Duchene et al. (2015) suggest that this procedure can be improved by randomising clusters of sequences sampled at the same time rather than individual taxa. Although such randomization approaches can demonstrate that the empirical sampling times are more informative than random sampling times, they can be very time consuming to compute for large data sets.

At present, the regression analysis in TempEst is based on a single phylogeny and therefore does not take into account uncertainty arising from phylogenetic estimation. To explore this uncertainty, multiple trees can be sampled from a phylogenetic posterior distribution or bootstrap distribution, each of which is then analysed separately in TempEst (see Gray et al. 2013 for an example). However, this approach will be automated in future versions of the software.
